# Cell Calcification Models and Their Implications for Medicine and Biomaterial Research

**DOI:** 10.1002/adhm.202501104

**Published:** 2025-09-27

**Authors:** Luke Hunter, Caterina Vanelli Coralli, Robert Dzhanaev, Aaron Morgan, Willi Jahnen‐Dechent, Sergio Bertazzo

**Affiliations:** ^1^ Department of Biomedical Engineering University College London London WC1E 6BT UK; ^2^ Institute of Cardiovascular Science University College London London WC1E 6BT UK; ^3^ Helmholtz‐Institute for Biomedical Engineering RWTH Aachen University Hospital 52074 Aachen Germany; ^4^ London Centre for Nanotechnology University College London London WC1E 6BT UK

**Keywords:** biomineralization, calcification, calcium, cell culture, dystrophic calcification, pathological calcification, phosphate, mineralomics

## Abstract

The evolutionary significance of calcium‐based minerals is highlighted by the division of life between those animals with backbones (vertebrates) and those without (invertebrates). Calcification, or biomineralization, is the process by which the tissues containing minerals are formed, occurring during normal physiological processes, such as bone remodeling, or in pathological conditions, like cardiovascular calcification. The 50th anniversary of the creation of the first true calcification cell culture model was in 2024, and these have become increasingly important to many fields of research in biology and medicine. Both the literature and experimental protocols associated with cell calcification models have become extremely fragmented across the many different research streams pursued. Here, it is aimed to give a comprehensive overview of the range of models available, and the approaches taken in these models, highlighting when and how methodological divergences arise, and identifying their most common issues. Ultimately, it is aimed to propose solutions to improve both replicability and the scientific relevance of such models. Cell models are fundamental to understanding pathologies where calcification is present and to improve knowledge of the most fundamental tissues in vertebrates: bones and teeth.

## Graphical abstract

Calcification, is the process by which the tissues containing minerals are formed, occurring during normal physiological processes, or in pathological conditions. Here, it is aimed to give a comprehensive overview of the range of cell models available, and the approaches taken by these models, highlighting when and how methodological divergences arise, and identifying their most common issues.

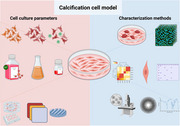

## 1 Introduction

Biomineralization or, as it is better known in medicine and to the public, calcification of soft tissues (see **Table** **1** for common terms related to calcification), is a much more common phenomenon than generally recognized. Calcification is the formation of an organic–mineral composite, naturally or in a pathological context, composed of an inorganic solid compound (mineral) in combination with cells, proteins, and extracellular matrix (ECM). In vertebrates, these calcified tissues and pathological calcification are typically formed from calcium‐phosphate‐based minerals, in contrast to invertebrates, which mostly form calcium‐carbonate‐based minerals.

**Table 1:** Related terms used in cell culture models of calcification.

Term	Definition
Calcification	The whole “entity/nodule/deposit/drusen/agglomeration” composed of a calcium phosphate mineral, with or without the presence of proteins, organic molecules, ECM, and cells, having a defined or undefined composition, morphology, or crystallinity.
Calcification process	The entire biological process or the result of a biological process in which, through a biological mechanism, there is precipitation of a calcium phosphate mineral, with or without association to proteins, organic molecules, ECM, and cells.
Metastatic calcification	Calcification that occurs in normal tissues as a result of high calcium levels in the blood (hypercalcemia).
Ectopic calcification	Calcification occurring in a region outside bone tissue, specifically in soft tissues.
Heterotopic ossification	The formation of bone in soft tissues where bone normally does not exist.
Dystrophic calcification	Calcification appearing on soft tissues that is not organized bone formation.
Ectopic bone	Bone tissue formed in soft tissue where it is not supposed to form.
Calcinosis	Abnormal calcium phosphate deposition in soft tissues.
Drusen	Term used in age‐related macular degeneration and retinal degeneration for calcification.
Extraosseous calcifications	Term used in nephrology for calcification.
Bone	Tissue formed with a characteristic hierarchical structure from the nanoscale to the macroscale, seen in many animals from from fish to humans. The nanostructure can be easily and clearly recognized by electron microscopy.^[^ ^1^, ^7^, ^19^ ^]^ Formed by ≈70% of calcium phosphate (a form of low crystallinity apatite), 20% collagen, and 10% other proteins.^[^ ^1^ ^]^
Mineral	A solid, inorganic substance present in calcification or other hard tissues, which may or may not present a specific phase or a mixture of phases (a mineral phase is a chemically and structurally homogeneous portion of the mineral in which all physical properties, such as crystallinity and composition, are the same). A mineral is generally formed by a single calcium phosphate phase or a mixture of calcium phosphate phases.
Mineralization	The process of precipitation of a mineral in a region or in/on ECM or cells.
Solution^[^ ^37^ ^]^	A gas, liquid, or solid phase containing more than one substance, in which one is the solvent (the major component of the solution) and the others are the solvents, and they are evenly distributed, forming a single phase.
Ionic solution	A solution that contains ions.
Solid	A solid has definite shape and volume and does not flow like liquids or gases under room temperature and pressure.
Particles	Any small fragment. It can be a solid, a part of a cell, or even vesicles.
Precipitation	Condensation of a solid from a solution.
Calcium	Calcium element or the metal calcium.
Calcium ions	Ions formed by ionized calcium atoms.
Calcium compounds	Compounds that contain calcium ions.
Calcium salts	A solid, containing calcium and at least one other element, with predominantly ionic bonds. It typically dissociates into constituent ions when dissolved in solution.
Apatite	A generic term for calcium phosphates with different crystallinities but a composition similar to hydroxyapatite.
Hydroxyapatite	Crystalline solid with the chemical formula Ca_5_(PO_4_)_3_OH.
Molecular mechanism	How molecules/proteins interact to produce a result.
Cellular mechanism	How cells interact to other cells, tissues or molecules/proteins to produce a result.
Ionic/precipitation mechanism	How ions interact to form a solid compound.

In healthy humans, the only tissues expected to calcify are bones, teeth, and the hair‐like cells of the inner ear.^[^ ^1^ ^]^ But the importance of calcification goes far beyond these. Calcification is associated with a wide range of diseases, many of which are leading causes of mortality worldwide including cardiovascular diseases,^[^ ^2^, ^3^ ^]^ cancer,^[^ ^4^, ^5^ ^]^ and even Alzheimer's disease.^[^ ^6^ ^]^

Biomineralization or “natural calcification” also offers a wellspring of design inspiration. The hierarchical structures of biomineralized tissues can span nine orders of magnitude with motifs unique to individual species.^[^ ^1^, ^7^ ^]^ The mantis shrimp, for example, wields supersonic‐shock resistant claws made of calcium phosphate.^[^ ^8^ ^]^ While eating, Port Jackson sharks’ calcium phosphate teeth self‐sharpen via architected wear.^[^ ^9^ ^]^ Such tissues offer inspiration for (bio)engineers and material scientists to create systems and materials with unique properties.^[^ ^10^ ^]^

Despite its importance, it is still unclear to the field how such variations in natural calcified tissues and pathological calcification occur or the cellular and biochemical mechanisms of formation of these tissues. Over the years, cell culture models have been widely used for basic research in this field, particularly of calcification in humans. Because calcification occurs across many diseases and tissues, cell calcification models extend beyond bone‐ and tooth‐associated cells. They exist for many organs in the body including the heart, blood vessels, teeth, the brain, the eye, the kidneys, the lungs, skin, blood, cartilage, and breast cancer.

**Figure 1:**
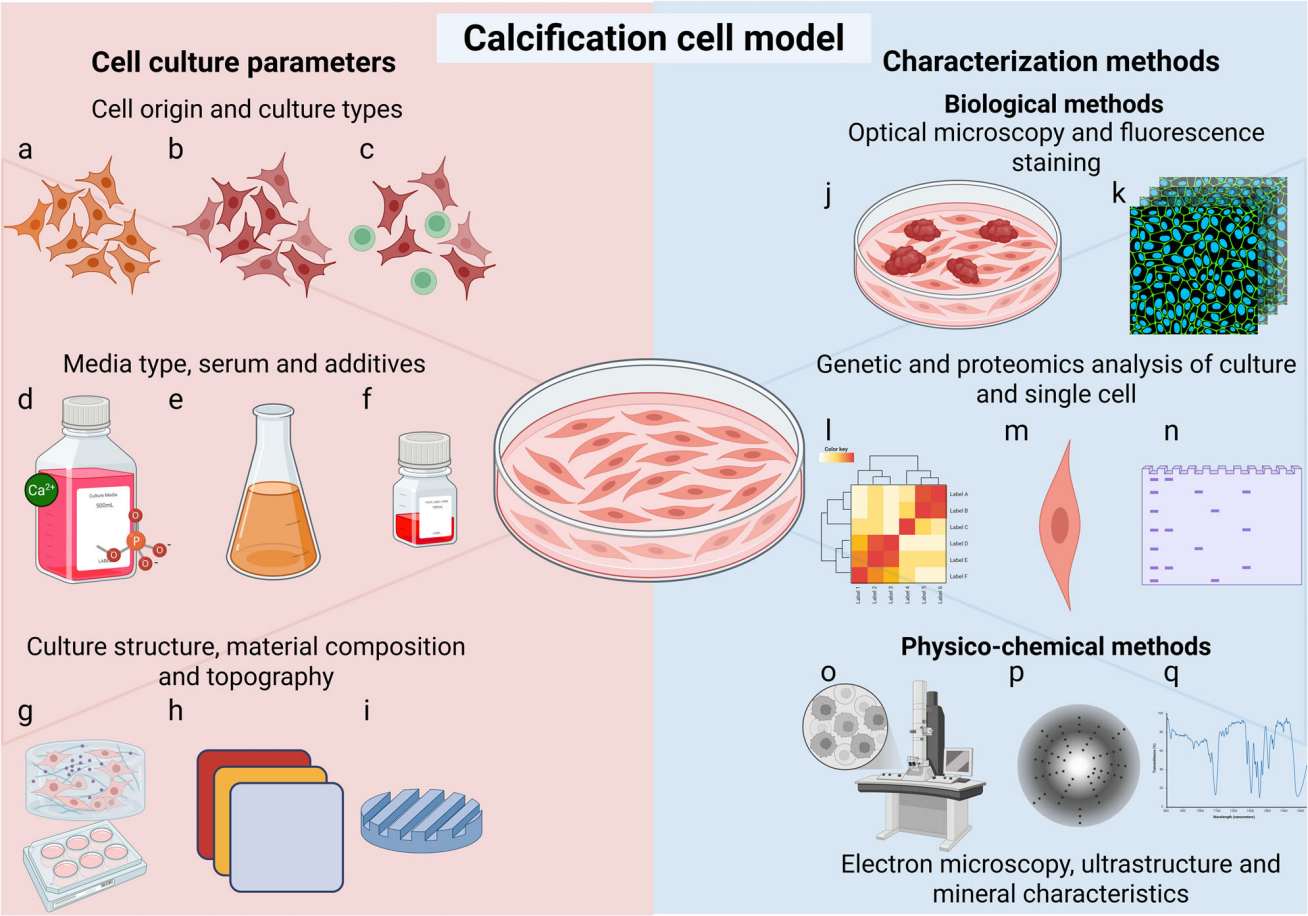
Calcification cell models typically utilize either a) homogeneous cell lines or b) heterogeneous cultures of primary cells. c) To study the influence of one cell type on another, deliberate cocultures of different cell lines or types can be used. d) Culture media selection is crucial, involving choices about the base medium, e) mineral supersaturation, source of mineral (inorganic or organic), amount, and quality of serum, f) antibiotics for contamination prevention, particulate matter as calcification promoters, and sometimes an inhibitor. g) Traditionally conducted on flat plastic surfaces, 3D substrates like collagen gels for cells to infiltrate better mimic ECM. h) The design and material of the substrate also play significant roles as the combination of i) topography and (h) material both determine substrate mechanical and chemical properties such as elasticity. j,k) Postculture, mineral and cell locations, and morphologies are typically analyzed using optical microscopy, with techniques like Alizarin Red or von Kossa staining to highlight calcified features. Detailed investigations into the calcification process involve live microscopy for longitudinal studies, fluorescent antibody staining, and confocal microscopy for specific cell structures. l,m) Cellular responses to calcification, such as differential expression and the analysis of gene regulatory networks are best examined by unbiased techniques like single cell sequencing including in their latest variants, spatial and time information. n) Protein expression is best studied by (space‐resolved) mass spectrometry and confirmed by immunoblotting. The composition and structure of the minerals formed may be analyzed using o) electron microscopy and p) diffraction, q) spectroscopy. Figure created with BioRender.com.

These calcification cell models serve multiple, sometimes conflicting objectives. Bone models aim to elucidate how this tissue forms, and biomaterial cell models for bone and tooth implants strive to promote healthy bulk calcification.^[^ ^11^ ^]^ Conversely, models of cardiovascular calcification seek to understand and prevent calcification in vascular tissue, while biomaterials for the replacement of soft tissue, such as bioprosthetic heart valvesstent, are designed to avoid calcification entirely.^[^ ^12^ ^]^ Therefore, these models are critical to modern medicine: they not only help elucidate how natural calcified tissues form but also support the development of treatments, biomaterials, and cures, which can involve encouraging or mitigating calcification (**Figure** **1**).

Notwithstanding this wide range of applications and objectives, such models are often grouped under the term “cell calcification models.” At first glance, this might suggest these models are producing the same organic compounds, ECM, and consequently the same inorganic calcification. Indeed, differentiation into an “osteoblast‐like” cell phenotype and thus a “professional mineralizer” is currently a common interpretation of why cells other than bone and tooth cells can also calcify. It seems counterintuitive though that “nonprofessional mineralizers” like smooth muscle cells, epithelial cells, and even neurons would all produce the same complex, hierarchically structured organic–mineral composite present in healthy bone, teeth,^[^ ^1^, ^13^ ^]^ and osteoderms.^[^ ^14^, ^15^ ^]^ Indeed, the mineral produced, and biochemical pathways observed during many types of pathological calcification resemble those observed in bone formation.^[^ ^16^ ^]^

However, similar arguments apply to teeth, a different tissue formed by distinct cells. The gross histological examinations and stains that are used to identify osteoblasts in calcified lesions^[^ ^16^ ^]^ respond in the same way to odontoblasts and the mineral they produce.^[^ ^17^ ^]^ The biochemical pathways that induce odontoblasts and osteoblasts to mineralize are very similar^[^ ^13^ ^]^ and the most common marker proteins used to identify an odontoblast phenotype^[^ ^17^ ^]^ cannot qualitatively distinguish between osteoblasts and odontoblasts.^[^ ^18^ ^]^

Despite these similarities, teeth and bones are certainly different. The same can be expected and is true of pathological calcification. Mineral produced during pathological calcification has different phase compositions,^[^ ^19^ ^]^ morphologies,^[^ ^3^ ^]^ and mechanical properties^[^ ^1^ ^]^ to bone. Further to this, cell models suggest that osteoblasts and vascular cells in calcification‐inducing media have different transcriptomic profiles,^[^ ^20^ ^]^ respond to inhibitors differently,^[^ ^21^ ^]^ and calcify to different extents.^[^ ^21^, ^22^ ^]^ A more recent addition to osteogenic cells, which mineralize physiologically (professional mineralizers) and cells like vascular smooth muscle cells, which frequently calcify pathologically (nonprofessional mineralizers) are ligament cells, e.g., periodontal ligament cells.^[^ ^23^ ^]^ These cells naturally mineralize at the bone or tooth junction yet remain unmineralized in the ligament part. This intriguing dual behavior is an important addition to calcifying models, as it may be particularly useful for studying the molecular mechanisms governing calcification or its prevention.

These difficulties in distinguishing cells and calcification mechanisms also occur in vitro. For example, the putative osteogenic process in a cell model is commonly suggested by the presence of osteoblast‐related proteins, such as runt‐related transcription factor 2 (Runx2), the sibling family protein secreted phosphoprotein 1 (commonly known as osteopontin, OPN), and the ECM component osteonectin (SPARC).^[^ ^17^ ^]^ However, these proteins are also found in various cell types, including sperm, epithelial cells, and neurons.^[^ ^24^, ^25^, ^26^ ^]^ Osteopontin is a prime example of a mineral‐binding protein found at the mineralized bone interface, hence its Latin denomination as “bone‐bridge,” and at most calcification sites. Yet, osteopontin, along with fetuin‐A, have been called “mineral chaperones.”^[^ ^27^ ^]^ Mineral binding alone does not qualify a protein as osteoblastic or osteoblastogenic, because many proteins are pleiotropic and participate in seemingly unrelated biological processes. Indeed, OPN is generally associated with tissue damage and remodeling including decidua formation.^[^ ^28^ ^]^ Still, many researchers regard OPN primarily as a “bone protein,” while others count it among cytokines and immunomodulators^[^ ^29^ ^]^ powerful enough to supplement baby formula.^[^ ^30^ ^]^ Moreover, cell model contamination by other cell types capable of calcifying, such as fibroblasts and stem cells,^[^ ^31^ ^]^ is common in primary cell cultures.^[^ ^32^ ^]^ As we will describe, multimodal characterization is necessary to confirm an osteoblast phenotype and bone formation in calcification models.

**Figure 2:**
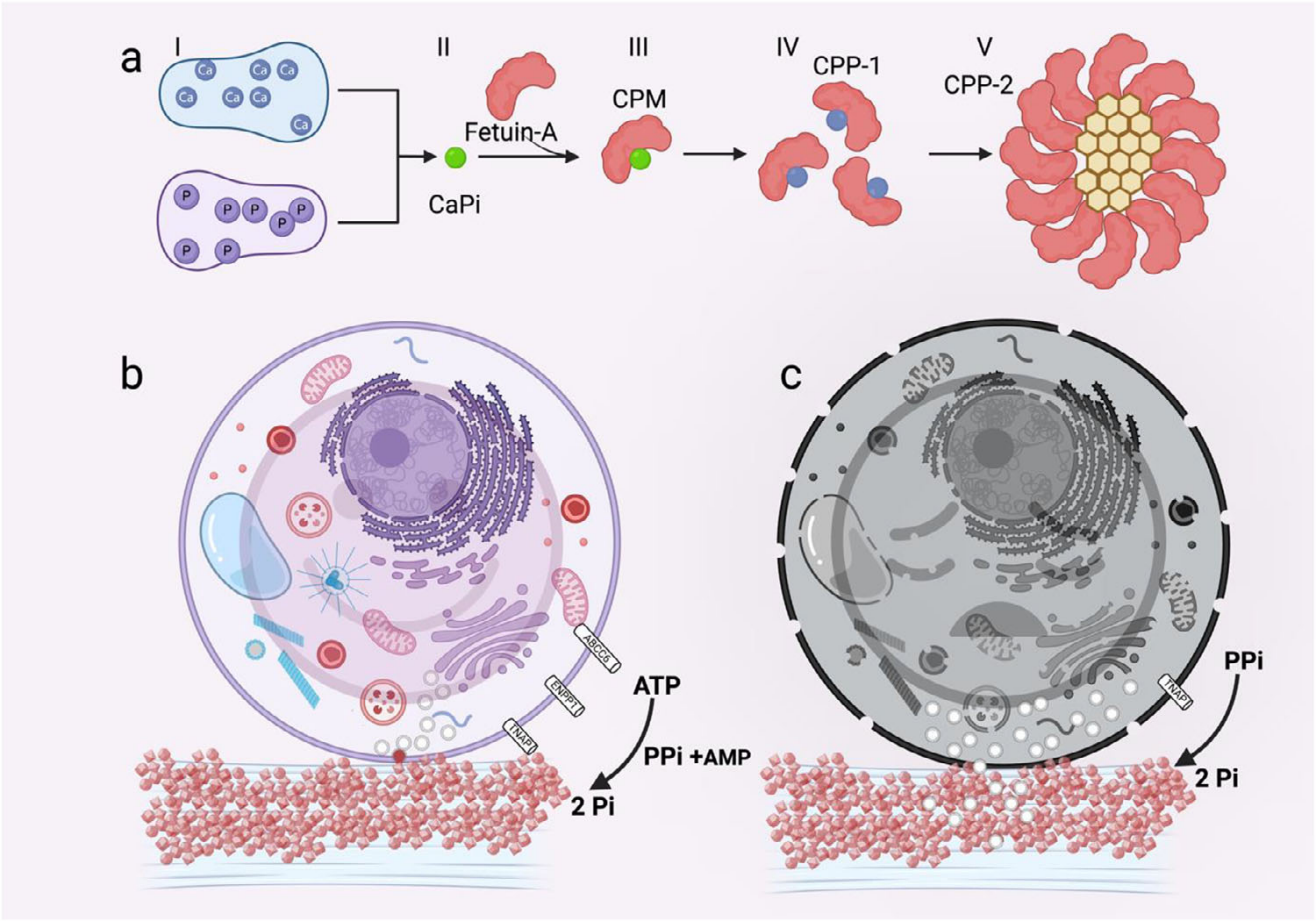
Proposed mechanisms of calcification in cell culture models. a) I) Calcification media typically comprise base medium (e.g., DMEM, M199), FBS, and increased Ca and P (or BGP, which will yield P upon phosphatase cleavage). II) Elevated Ca and P immediately form crystal precursors, which must be stopped from growing to prevent precipitation. III) In physiological fluids mineral‐binding proteins like fetuin‐A, (the major globulin in FBS) can form tight interaction with these ion clusters, chaperoning mineral through nucleation, phase separation, and eventual crystallization. The protein–mineral complexes stabilized by fetuin‐A are called CPPs. The building blocks for CPP are calciprotein monomers (CPMs), composed of CaP ion clusters tightly bound and stabilized by monomeric fetuin‐A. IV) CPM can consolidate into spherical 30–100 nm aggregates (primary CPP; CPP‐1), carrying insoluble mineral as amorphous calcium phosphate in diffuse fetuin‐A‐bound mineral domains. V) In a final step, the mineral phase can undergo transition and rearrangement into ordered, more thermodynamically stable crystalline phases like hydroxyapatite (secondary CPP; CPP‐2), where the mineral is organized in densely packed needle‐shaped lamellae. Morphologically, these distinctive crystalloid secondary CPPs take the form of prolate ellipsoids, with long axis diameters of 100–250 nm. Approximate sizes are shown. b) Physiological mineralization cell model versus c) pathological calcification cell model. The chemical precipitation of calcium phosphate from the ionic precursors is the final step in both physiological bone mineralization and pathological calcification. The ionic environment and the sum of activators and inhibitors regulate this chemical precipitation. (b) In bone cell calcification models, transcription factors control the differentiation of mesenchymal precursor cells into osteoblasts. The osteoblasts typically secrete adenosine triphosphate (ATP), through the ATP‐binding cassette subfamily C member 6 (ABCC6) transporter, cleave the ATP into pyrophosphate (PPi) and AMP through ectonucleotide pyrophosphatase/phosphodiesterase 1 (ENPP1), and cleave PPi into two phosphates (2 Pi) through TNAP. After removal of PPi as an inhibitor, calcification of ECM and cellular debris (vesicles and cell remnants) will take place. Mineral chaperones such as fetuin‐A stabilize calcium and phosphate as CPPs. CPPs stabilize supersaturated mineral in the extracellular space and during transcellular transport. (c) In pathological cell calcification models, stressed or dead cells produce little ATP and therefore lack PPi, a potent inhibitor of cell calcification. Stressed, apoptotic, and necrotic cells release extracellular vesicles, cell debris, and leaky mitochondria (which are rich in Ca), all of which present crystallization nuclei that readily calcify along with any ECM. Figure created with BioRender.com.

Given the persistent uncertainties surrounding cell calcifying models, a fundamental question arises: does the cell model being used reproduce, at all, the intented biological system? For example, does a human bone calcification model truly replicate bone mineralization? Does a smooth‐muscle‐cell‐based cardiovascular calcification model recapitulate any relevant features of the disease? Perhaps more importantly, to what extent is calcification driven by cellular activity versus the effects of culture media, which may induce mineralization even without viable cells (**Figure** **2**). In other words, is the model inducing calcification under artificial conditions that bear little resemblance to the in vivo biology it aims to represent? Surprisingly, these questions are rarely posed when designing cell‐based calcification models and thus remain unaddressed in much of the published literature. For example, medications that perform well in cell models of osteoporosis^[^ ^33^ ^]^ or cardiovascular calcification^[^ ^34^, ^35^, ^36^ ^]^ often fail in clinical trials as a consequence of these unresolved questions.

In this review, we aim to clarify what is known, what remains unknown, and most importantly highlight potentially misleading assumptions in current research that use cell calcification models. Our goal is to support progress in biology, medicine, and bioengineering by outlining critical considerations for using these models and suggesting best practices for conducting experiments and reporting results. Cell calcification models are fundamental not only for studying several diseases, but also for understanding how animals produce calcified tissues—a cornerstone of our comprehension of a vital aspect of life and necessary knowledge for the development of high‐performance biomimetic materials.

### 1.1 The First Calcification Models—Bone

In 1964, Peck et al. demonstrated for the first time a method for culturing healthy bone cells that survived long periods.^[^ ^38^ ^]^ Although cells remained healthy, calcification was not observed. It was not until 1974 that a primary bone cell culture produced calcium phosphate mineral.^[^ ^39^ ^]^ Binderman et al. cultured primary bone cells from rat periosteum in a culture medium containing ≈2 mm of dissolved sodium and potassium phosphate, changing every 3–4 days, for 2 months.^[^ ^39^ ^]^ Phosphate, indicating the presence of calcium phosphate minerals, was revealed with von Kossa staining. Additionally, transmission electron microscopy revealed spiky, electron‐dense laths collocated with nonelectron dense collagen, indicating that some collagen fibrils had calcified.

It was later suggested that bone cells would only mineralize when there was high levels of phosphate in the culture medium.^[^ ^40^ ^]^ This discovery gave rise to the idea that the culture medium required a specific additive to induce cell calcification in vitro. As a result, various methods of increasing phosphate levels in the medium were tested, with the organic phosphate compound β‐glycerophosphate (BGP) emerging as a convenient inducer of calcification,^[^ ^41^ ^]^ which does not precipitate with medium calcium on its own, but needs to be cleaved by tissue nonspecific phosphatase (TNAP), a crucial enzyme of calcification in vivo, to liberate ionic phosphate necessary for mineral formation.^[^ ^42^, ^43^ ^]^ Current recommendations suggest using 2–10 mm BGP for calcification models, depending on the phosphatase activity of a given cell line. Cells lacking sufficient phosphatase activity do not calcify in these conditions, and cells with strong inherent phosphatase activity like SaOS‐2 cells do not tolerate high BGP, because the high amounts of phosphate liberated are cytotoxic and can lead to the simple precipitation of supersaturated calcium phosphate mineral in the medium.^[^ ^44^, ^45^ ^]^ Phosphatase secretion is a common “differentiation marker” and indeed a hallmark of the senescence associated secretory phenotype of aged cells and therefore senescent cells tend to calcify.^[^ ^46^ ^]^ In addition to BGP, ascorbic acid and dexamethasone are routinely used in calcification models,^[^ ^47^ ^]^ collectively referred to as osteogenic induction or differentiation medium. Ascorbic acid is a cofactor of proline hydroxylase that allows cells to produce more collagen by hydroxylating proline and lysine to form procollagen.^[^ ^48^ ^]^ Dexamethasone is a synthetic glucocorticoid hormone derivative that induces widespread transcriptomic changes, including the upregulation of Runx2, a protein that regulates osteoblast proliferation and differentiation in mesenchymal cells.^[^ ^49^ ^]^ Following these pivotal discoveries, more complex cell calcification models have been developed.

## 2 Healthy Cell Calcification Models

### 2.1 Cell Calcification Models—Bone

Contemporary cell calcification models have advanced well beyond basic static cultures supplemented with osteogenic additives. For example, miniaturized “lab‐on‐a‐chip” systems have been developed to replicate the dynamics of a bone remodeling microenvironment. These microfluidic platforms use small quantities of cells and culture media while enabling the application of mechanical forces, often via controlled media perfusion,^[^ ^50^, ^51^ ^]^ to simulate the stimuli that drive osteocytes to regulate bone formation.

In 2023, a “bone‐on‐a‐chip” model successfully induced an osteoblast–osteoclast coculture to generate a calcified trabeculae‐like organoid several hundred micrometers in size, without relying on a significant cellular scaffold.^[^ ^51^, ^52^ ^]^ This is in contrast to conventional 2D cultures, where such self‐assembled structures do not form, and to 3D scaffold‐based cultures, where prefabricated trabecular substrates inhibit observation of the self‐assembly of new trabeculae,^[^ ^53^, ^54^ ^]^ a process where cells organize themselves into structures that are thought to underpin early stage bone formation and remodeling.^[^ ^51^ ^]^ Such osteoblast–osteoclast cocultures are particularly relevant as they more accurately reflect physiological conditions, where osteoclasts create a conducive microenvironment and actively recruit osteoblast progenitors to facilitate bone remodeling and mineralization.^[^ ^55^ ^]^

Another recent and important change to cell calcification models is that the substrate has evolved from simple tissue culture plastic to biomimetic hydrogels, notably porous and compliant gels made from natural polymers like collagen^[^ ^56^, ^57^ ^]^ and silk.^[^ ^53^ ^]^ Indeed, the first cell calcification models with a substrate other than glass or plastic used collagen gels.^[^ ^57^ ^]^ These models were also among the first to be conducted on 3D, organoid‐like structures rather than flat culture dishes. In these 3D cultures, osteoblast progenitor cells infiltrated and calcified the gel when exposed to BGP. These gels are now used for culturing most of the 3D cell models able to create organoids. These cells can remodel the culture gels similarly to native ECM,^[^ ^56^ ^]^ and mineralization may be further encouraged through tuning the substrate stiffness^[^ ^58^ ^]^ or adding calcification inductive proteins and particles, such as fibronectin^[^ ^51^ ^]^ or graphene oxide.^[^ ^54^, ^59^, ^60^ ^]^ Despite the evolution of bone models, most modern cell calcification models still rely on additives, such as BGP, to induce differentiation or calcification.^[^ ^50^, ^51^, ^53^, ^54^ ^]^

All these new methods and materials have significantly expanded the scope of bone cell calcification models, moving beyond the simple understanding of how bone cells produce minerals (though this remains an area of ongoing exploration). Contemporary models are investigating potential new calcification mechanisms^[^ ^61^ ^]^ and are being used to study diseases involving bone and unrelated cell types, such as neutrophils.^[^ ^62^ ^]^ Moreover, the development of more complex and in vivo‐like organoids has provided deeper insights into the interactions between bone cells and implants.^[^ ^63^ ^]^ Finally, modern bone cell cultures have been instrumental in the study of de novo self‐assembly of bone structures such as trabeculae.^[^ ^51^ ^]^

### 2.2 Cell Calcification Models—Implants

Cell calcification models to assess the biocompatibility and various effects of implant materials gained popularity once conditions for sustaining healthy, mineralizing cultures were established. Indeed, shortly after the first experimentations with collagen gels^[^ ^57^ ^]^ (following the establishment of the glass and plastic culture methods), models incorporating ceramic^[^ ^64^ ^]^ and metal^[^ ^65^ ^]^ substrates emerged. Employing these newly established cell models, primary bone cells were cultured directly on ceramics known to induce bone formation in animal models such as calcium phosphate bioceramics and silica–calcium bioglasses.^[^ ^66^ ^]^ Importantly, from the perspective of understanding the triggers of cell calcification, calcification occurred in the absence of media additives such as BGP.^[^ ^64^, ^67^ ^]^ By contrast, calcification on other materials also used in bone implants, such as titanium, required the addition of media supplements to primary bone cell cultures.^[^ ^65^ ^]^ These results, even if not unexpected since the ceramics were known to induce bone formation when implanted, highlight key factors influencing a cell's capacity to calcify.

First, the substrate can be important for cell behavior. An inorganic substrate can induce bone cells to calcify even in the absence of hormone or growth factor stimulation, a situation called “osteoinductive.” This does not happen when the same cells are cultured on the main ECM component of bone collagen. Second, the material's stiffness (elastic modulus) influences calcification, though similarly stiff substrates do not necessarily yield the same outcome. The ability of bioceramic and bioglass substrates to consistently support calcification without media additives, such as BGP, appears unique in biomaterials and suggests a driving factor for bone cell calcification is the availability of calcium and phosphate ions (provided by the dissolution of the ceramics or additives). These results highlight the profound effect of inorganic components on cell behavior. Beyond advancing the fundamental understanding of calcification, these models are also valuable in bioengineering, particularly when mineralization is desired, like in bone implants, but also in developing cardiovascular and ocular implants that must not calcify, like heart valves or intraocular lenses.

The evolution of substrates in cell calcification models mirrored findings from animal models and clinical practice. Flat metal surfaces were gradually replaced with porous substrates,^[^ ^68^ ^]^ often coated with ceramic,^[^ ^69^ ^]^ as these materials became increasingly common in clinical settings.^[^ ^70^ ^]^ At the same time, while stiff metals and ceramics were widely used in orthopedic clinical practice to replace load bearing bone, the idea of an organic soft substrate to induce bone formation had also been evolving in parallel to the use of gels in the cell calcification research setting. While acellular, demineralized bone ECM became a popular bone void filler,^[^ ^71^ ^]^ synthetic gels designed to mimic the ECM became increasingly used substrates in calcification models.^[^ ^72^ ^]^ As expected, these advancements led to the testing of new material combinations, including 3D‐printed apatite^[^ ^73^ ^]^ and gels infused with apatite particles.^[^ ^74^ ^]^

Despite the success in inducing bone formation by implants combining ceramics and other materials, or simply soft scaffolds able to induce osteogenesis, there remained an unmet clinical need: the demand for large, mechanically stable bone implants. Such implants are typically metal, as this is the only material class with the mechanical properties necessary for load‐bearing applications. Nevertheless, researchers aimed to tailor the implant surface to induce bone cells that calcify and tightly integrate with the host bone. Thus, cell calcification models studied materials, cell culture additives, as well as chemical and physical characteristics of the surface and their impact on bone cell behavior.

Surface engineering of implant materials can dramatically influence calcification. For instance, calcium phosphate substrates, and to a lesser extent metals, typically promote both mineral calcification and osteoblast‐related protein expression more than plastic.^[^ ^59^, ^75^ ^]^ This could be due to ion exchange between the substrate and the culture media. Indeed in acellular cultures^[^ ^76^ ^]^ and cell cultures,^[^ ^77^ ^]^ some calcium phosphate minerals release phosphate into the medium while others deplete it, with the general trend being that culturing on apatite encourages cell calcification.

Beyond changes in material chemistry, substrate topography has a profound impact on calcification. As rough surface textures are typical of bone implant surfaces, particularly for metal implants, engineered roughness has been studied in cell calcification models. Mean deviation from the surface profile, the most common measure of substrate roughness, of 2.5–4 µm has been suggested to be optimal for calcification and osteoblast‐related protein expression on silica substrates.^[^ ^78^ ^]^ Above this threshold on silica and titanium substrates, calcification appears to decrease.^[^ ^78^, ^79^ ^]^ However, some work suggests that microroughness does not change the expression of osteoblast‐related proteins^[^ ^80^ ^]^ or that osteoblast‐related protein expression does increase but calcification does not^[^ ^81^ ^]^ when cultured on titanium substrates. In some cases, microroughness even appears to reduce calcification, such as on calcium phosphate–plastic composites.^[^ ^82^ ^]^

Roughness does more than simply increase the surface area available for ion exchange, as increasing roughness does not necessarily increase substrate dissolution.^[^ ^83^ ^]^ Indeed, precise topographical changes to substrates traditionally used in control cultures because of their known inability to induce calcification, can induce cells to express osteoblast‐related proteins and calcify. These observations have been attributed to cell mechanotransduction, though the precise signaling pathways and causes are unknown.^[^ ^84^, ^85^ ^]^ A semirandom array of nanoscale groves on near‐atomically flat plastic caused stem cells to express osteoblast‐related proteins in the absence of media additives, whereas regularly arranged and fully random arrays did so less.^[^ ^84^ ^]^ Single‐cell cultures in half–half osteogenic and adipogenic culture media showed that a cell‐sized plastic platform in the shape of a square or triangle, but not a circle, can induce stem cells to express osteoblast‐related proteins.^[^ ^86^ ^]^

Cell calcification can also be induced by topographical changes to granular substrates. Plastic microdisks with nanoscale groves induced the calcification of stem cells cultured on flat plastic in the absence of osteogenic media, whereas smooth microspheres did not.^[^ ^85^ ^]^ These results show that both substrate chemistry and topography can have profound effects on cell calcification. Whether or not these microstructured surfaces will survive a clinical setting including blood, clotting, and immune cells, and still direct cell differentiation is currently unknown. Long‐term performance studies of soft implant materials with and without intricate biointerface design suggest that blood obliterates most engineered and finely tuned surfaces resulting in the well‐known “blood compatibility catastrophe” of biomaterials.^[^ ^87^ ^]^

## 3 Pathological Cell Calcification Models

Pathological calcification is a common consequence of many degenerative diseases, causing soft tissues to accumulate minerals. The most common mineral found in these calcifications is hydroxyapatite‐like calcium phosphates containing carbonate, magnesium, and other ion substitutes that affect it chemically stability at different pH. High‐resolution analyses have revealed the presence of multiple calcium phosphate phases.^[^ ^19^, ^88^ ^]^ However, in most cases, calcification occurring in extracellular spaces exposed to blood primarily consists of carbonate‐substituted basic calcium phosphates.^[^ ^1^ ^]^ Acidic calcium phosphate phases typically form in tissues without direct blood contact, like limb joint spaces.^[^ ^1^, ^89^ ^]^ Despite these observations, the mechanisms and cells responsible for these calcifications remain largely unknown, regardless of the associated disease. Furthermore, the phenomenon is so poorly understood that it is unclear whether calcifications across different tissues and various diseases share any mechanistic similarities or whether they are reversible at all.

The most common and prominent examples of pathological calcification are arguably kidney stones,^[^ ^90^ ^]^ affecting 2–20% of the population^[^ ^91^ ^]^ and vascular calcification, which develops in around 25% of adults.^[^ ^2^ ^]^ Cardiologists and vascular biologists have long argued that vascular calcification may have beneficial effects, such as contributing to the stabilization of atherosclerotic plaques.^[^ ^92^ ^]^ However, at earlier stages of disease, microcalcifications are generally considered detrimental, as they are strongly associated with plaque instability and rupture.^[^ ^93^ ^]^ Beyond these well‐known cases, pathological calcification has also been studied in a wide range of tissues using cell models derived from the brain,^[^ ^94^, ^95^ ^]^ teeth,^[^ ^17^ ^]^ eye,^[^ ^96^ ^]^ lung,^[^ ^97^, ^98^, ^99^ ^]^ skin,^[^ ^98^, ^99^, ^100^, ^101^, ^102^ ^]^ blood,^[^ ^99^ ^]^ cartilage,^[^ ^89^, ^103^ ^]^ breast,^[^ ^104^ ^]^ skeletal muscles,^[^ ^99^ ^]^ stomach,^[^ ^99^ ^]^ colon,^[^ ^99^ ^]^ prostate,^[^ ^99^ ^]^ cervix,^[^ ^99^, ^105^ ^]^ umbilical cord,^[^ ^98^ ^]^ kidneys,^[^ ^106^, ^107^, ^108^ ^]^ and amniotic membrane.^[^ ^98^ ^]^

### 3.1 Cell Models of Renal Calcification

The kidney, as an organ where a mineral‐rich fluid is being concentrated and excreted, is often affected by various types of ectopic calcification. The first demonstration of biological mineralization by renal cells in vitro used an epithelial cell line, which formed calcium phosphate microliths at their nonluminal surface after prolonged culture in standard media,^[^ ^109^ ^]^ consistent with an early stage of the “fixed‐particle” mechanism of renal calcification.

Urolithiasis, or the formation of stones (calculi) in the urinary tract, commonly occurs in the kidney (nephrolithiasis) and is mainly influenced by factors such as dehydration, dietary habits, metabolic disorders, and genetic predisposition.^[^ ^110^ ^]^ Kidney stones are typically composed of a nucleus of calcium phosphate covered by calcium oxalate.^[^ ^111^ ^]^ Two primary mechanisms of kidney stone formation have been proposed.^[^ ^111^ ^]^ The first is the fixed‐particle mechanism. Here, calcium phosphate nanoparticles in the renal papillae grow into large stone‐like masses known as Randall's plaques,^[^ ^112^, ^113^ ^]^ these plaques then erode through to the renal calyx. Oxalate can then deposit on top, forming a large stone which may then dislodge.^[^ ^114^ ^]^ The second mechanism is via the “free‐particle” mechanism where crystals form within the kidney tubules, aggregate into larger particles, and then block ducts.^[^ ^114^ ^]^ This creates a stony mass, known as Randall's plug, which can grow larger as more particles are deposited.^[^ ^114^ ^]^ This mechanism is believed to be primarily operative for patients with systemic disorders of calcium metabolism.^[^ ^114^ ^]^

The initial formation of mineral particles, particularly in the free‐particle mechanism, is thought to result from precipitation in the supersaturated urine of patients, often due to abnormal calcium and phosphate concentrations.^[^ ^114^ ^]^ Indeed, drugs that do not affect mineral supersaturation appear ineffective in reducing kidney stone formation.^[^ ^111^, ^115^ ^]^ Kidney cell cultures with elevated ion concentrations effectively mimic this supersaturation mechanism, reliably precipitating calcium‐based minerals.^[^ ^116^ ^]^ Other works using similar high calcium media have suggested nonosteogenic pathways to calcification, though did not confirm the presence of mineral through mineral‐specific characterization methods.^[^ ^117^ ^]^ Results obtained in this way should be interpreted carefully (see Section 4.4). As well as calcium phosphate, oxalate stone nuclei form spontaneously too and grow in synthetic urine, and microfluidic devices that reproduce flow in complex duct‐like geometries are enabling detailed studies of how flow, along with urinary inhibitors and promoters, influences the physicochemical pathways of crystallization.^[^ ^118^, ^119^ ^]^

By contrast, the fixed‐particle mechanism is more challenging to replicate due to the incomplete understanding of its processes. Proteins typically associated with osteoblasts, though not exclusive to them, are upregulated in tissues associated with nanoparticle formation, suggesting that an osteoblast‐like phenotype has a role in plaque formation.^[^ ^120^ ^]^ To model this, kidney cell calcification systems have incorporated osteogenic additives, such as BGP, to induce an osteoblast‐like phenotype and calcification.^[^ ^106^ ^]^ These models have proven invaluable for identifying calcification promoters, such as osteomodulin, which induces calcification in a dose‐dependent manner in vitro.^[^ ^106^ ^]^ Silencing osteomodulin in mice significantly reduces Randall's plaque formation, underscoring its potential as a therapeutic target.^[^ ^106^ ^]^

Despite these advances, significant gaps remain in cell models, as macrophage and fibroblast infiltration are commonly found in calcified papillae tissues, suggesting substantial immune involvement.^[^ ^120^ ^]^ Apoptotic bodies are also believed to play a critical role, and free‐particle and fixed‐particle calcification can occur simultaneously.^[^ ^114^ ^]^

Nephrocalcinosis is defined as the pathological deposition of calcium salts within the renal parenchyma, resulting in calcification of kidney tissue.^[^ ^121^ ^]^ This condition can develop due to various underlying metabolic disorders, elevated serum or urinary calcium levels (hypercalcemia or hypercalciuria), and is often associated with nephrolithiasis, where the formation of kidney stones may occur concurrently. However, some suggest that these conditions should be considered independent disorders with distinct pathophysiologic mechanisms.^[^ ^122^ ^]^

To better study these processes, advanced organoid cultures are being developed.^[^ ^107^ ^]^ These combine features of single‐mechanism models, such as investigations on stem cell differentiation into immune cells in calcium oxalate environments^[^ ^108^ ^]^ and osteoblast‐like phenotype induction^[^ ^106^ ^]^ into multimechanism systems.

### 3.2 Cell Models of Vascular Calcification

Cardiovascular calcification is diverse, affecting many anatomical sites: heart valves,^[^ ^123^ ^]^ the inner layer of aortic vessels (intima calcification), and the medial layer of aortic vessels.^[^ ^3^, ^124^, ^125^, ^126^ ^]^ It has been predominantly studied in vitro through valve interstitial cells and vascular smooth muscle cells, which are suggested to transdifferentiate into osteoblast‐like cells.^[^ ^127^ ^]^ This transdifferentiation is widely reported but is now considered a late‐stage event following extensive cell and tissue damage including the initial hardening of ECM by calcium phosphate deposition.^[^ ^128^ ^]^

The first reliable method for inducing both calcification and an osteoblast‐like phenotype in cardiovascular cell culture was via 10 mm BGP and 50 µg mL^−1^ ascorbic acid.^[^ ^129^ ^]^ This method, later refined to use 10 nm dexamethasone, is now widely used.^[^ ^130^ ^]^ Additionally, colloidal protein–mineral complexes called calciprotein particles (CPPs)^[^ ^131^ ^]^ can induce an osteoblast‐like phenotype and calcification in vascular cells even without osteogenic media.^[^ ^132^, ^133^ ^]^ Extracellular vesicles from bone cells can also cause vascular cells to express osteoblast‐related proteins and calcify more in cultures with osteogenic additives.^[^ ^134^ ^]^

Conventional 2D monoculture systems are widely used but more recently 3D culture systems, primarily based on gel substrates, are being adopted as they can more accurately mimic the in vivo microenvironment's topography, heterogeneity, and stiffness. Indeed, cell calcification models using such gels yielded cell protein expression networks with greater similarity to those of cells from explanted calcified valve tissue than to cells cultured on plastic.^[^ ^135^ ^]^ Coculture systems, such as those containing valve interstitial cells and endothelial cells, have been developed to better mimic heterogeneity and to probe cell–cell crosstalk.^[^ ^136^ ^]^ In these setups, endothelial nitric oxide consistently reduces activation and calcification in the neighboring interstitial cells.^[^ ^136^ ^]^ These close proximity models complement in vitro studies of longer‐range communication, including signaling via bone‐derived extracellular vesicles.^[^ ^134^ ^]^

Explanted ECM can mimic tissue microenvironments even more closely. Under quasiphysiological flow and pressure conditions, decellularized ECM has been used to examine how hemodynamics affect physicochemical calcification in aortic‐valve geometries, with mineral preferentially forming at high stress regions, as seen in vivo,^[^ ^12^ ^]^ and with calcification occurring to a greater extent than in glutaraldehyde‐treated bioprosthetic valves.^[^ ^137^ ^]^ Alternatively, explanted tissue and organ‐culture models, that largely preserve original cell populations and ECM, have been used to evaluate candidate therapeutics such as adenosine which reduced calcification in a dose‐dependent manner, as with in vivo observations, and unlike 2D cultures, these preparations allow region‐specific assessment of mineral and osteoblast‐like marker accumulation.^[^ ^138^ ^]^

Together, these models attempt to recapitulate vascular calcification, a complex process influenced by phosphate levels, oxidative stress, extracellular vesicles, and inflammatory mediators with osteogenic transdifferentiation occurring as a rare, late‐stage phenomenon. It should be stressed that most soft tissue calcification is dystrophic and typically associated with cell and tissue damage and defective remodeling,^[^ ^139^ ^]^ but is not associated with chondro‐osteogenic cell differentiation. Heterotopic ossification is a notable exception that is either caused by genetic mutations of the osteogenic activin receptor‐like kinase 1 signaling pathway^[^ ^140^ ^]^ or by iatrogenic dissemination of bone chips and thus bone‐morphogenetic‐protein‐rich matrix into surrounding soft tissue during orthopedic surgery, or catastrophically in blast wounding.^[^ ^141^ ^]^

While these mechanisms have been widely studied, significant gaps remain in the understanding of precise triggers of transdifferentiation in vivo and the interplay of various factors during calcification. Current experimental models have advanced our knowledge, but some results diverge from those found under in vivo conditions, and clinical trials targeting putative key calcification pathways have had limited success.

### 3.3 Other Cell Models of Pathological Calcification

Other than cardiovascular disease, pathological calcification is a feature of numerous conditions with significant public health impact. While renal and vascular calcification are a primary focus, models of calcification in other tissues, such as joints, breast, brain, lung, skin, and eye, have provided critical insights into disease mechanisms and potential therapies.

#### 3.3.1 Osteoarthritis and Joint Calcification

Osteoarthritis, affecting over 500 million people globally and a leading cause of disability,^[^ ^142^ ^]^ is often worsened by calcified particles in the joints. Cartilage chondrocytes transdifferentiating into osteoblast‐like cells exacerbate this pathology.^[^ ^143^ ^]^ Cell models of osteoarthritis have typically used primary cartilage cells from patients, which spontaneously calcify in standard culture media.^[^ ^89^ ^]^ More recently, tissue culture models have been investigated and have, for example, shown tissue degradation and inflammation markers, such as cytokines, increase in cultures with osteogenic additives compared to those without,^[^ ^103^ ^]^ reflecting observations in vivo that calcification induces an inflammatory response and cartilage damage.^[^ ^143^ ^]^ However, the in vivo relevance of such models should be considered carefully as they used extremely high BGP concentrations (20 mm),^[^ ^103^ ^]^ which likely caused nonphysiological levels of phosphate in the culture and simple precipitation of calcium phosphate.^[^ ^44^, ^45^ ^]^

#### 3.3.2 Breast Cancer Calcification

Breast cancer is the most common cancer among women, with over 2.3 million new cases diagnosed annually.^[^ ^144^ ^]^ Apatite and calcium oxalate calcifications are frequently found in breast cancer, with apatite presence potentially indicating more serious tumors.^[^ ^4^ ^]^ Bone‐associated proteins are concentrated near calcified deposits in biopsies.^[^ ^145^ ^]^ To investigate this, 2D mammary cell models have been developed that aimed to induce an osteoblast‐like phenotype using BGP, ascorbic acid, and dexamethasone.^[^ ^104^ ^]^ More recently, 3D organoid models have been developed and, for example, have been used to investigate the effects of different minerals on breast tumor cells, showing apatite promotes an aggressive proliferative pathway whereas oxalate suppresses it.^[^ ^146^ ^]^ These models have provided insight into the role of calcification in cancer progression and its diagnostic potential.

#### 3.3.3 Neurological Disorders and Brain Calcification

Dementia, a neurodegenerative disorder, affects around 60 million people worldwide,^[^ ^147^ ^]^ with around 30% exhibiting calcification large enough to be visible with X‐ray scans.^[^ ^148^ ^]^ The basal ganglia are known to be particularly susceptible to calcification^[^ ^6^, ^148^ ^]^ and osteopontin has been detected in calcified lesions.^[^ ^149^ ^]^ Recent calcification models of the ganglia have since applied BGP, ascorbic acid, and dexamethasone to induce an osteoblast‐like phenotype and calcium phosphate mineral production to study the effects of therapeutic interventions such as the hormone calcitriol.^[^ ^94^, ^95^ ^]^

#### 3.3.4 Calcification in the Lungs and Skin

Chronic respiratory diseases, including those involving lung injury and calcification, are responsible for around 4 million deaths annually.^[^ ^150^ ^]^ Pulmonary calcification is a well‐documented consequence of tuberculosis,^[^ ^151^ ^]^ often developing after caseation necrosis of granulomas. Calcification is believed to encapsulate and isolate uncleared infectious mycobacteria^[^ ^152^ ^]^ and thus opaque lesions in chest X‐ray screenings are a major diagnostic criterion to identify tuberculosis carriers.

A rare but informative condition, pulmonary alveolar microlithiasis (PAM), is characterized by the accumulation of spherical calcium phosphate deposits in lung tissue. PAM is caused by mutations in the gene encoding sodium‐dependent phosphate cotransporter 2B.^[^ ^153^ ^]^ Even though injured lung tissue often mineralizes,^[^ ^151^ ^]^ the corresponding mechanisms and prevention strategies are comparatively understudied through in vitro models compared to other diseases that cause pathological calcification. An osteogenic pathway has been suggested, based on observations such as Runx2 upregulation and apatite in calcified tissue,^[^ ^151^ ^]^ and tracheal cell cultures have been induced to calcify in the presence of osteogenic culture medium with BGP, ascorbic acid, and dexamethasone.^[^ ^97^ ^]^

Similar approaches have been applied to study pathological calcification in the skin, particularly in the context of chronic inflammatory and connective tissue disorders such as pseudoxanthoma elasticum.^[^ ^100^, ^154^ ^]^ 2D skin cell cultures supplemented with osteogenic media readily calcify.^[^ ^100^, ^154^ ^]^ These models have identified potential therapeutic interventions, including the inhibition TNAP, as a means to reduce ectopic calcification.^[^ ^100^, ^154^ ^]^ Notably, inhibiting TNAP in mice did not affect healthy bone remodeling.^[^ ^154^ ^]^

#### 3.3.5 Calcification in the Eyes

The progression of age‐related macular degeneration (AMD), a disease that eventually causes blindness and affects over 200 million people worldwide,^[^ ^155^ ^]^ is closely associated with the formation of calcified drusen deposits in the retina but not with an osteoblast‐like phenotype.^[^ ^156^ ^]^ A recent retinal epithelium cell model replicated drusen formation with realistic morphology and chemical composition, notably without requiring BGP, ascorbic acid, or dexamethasone.^[^ ^96^ ^]^ Building on this model, others have investigated drusen formation over extended culture periods, using periodic staining and in situ fluorescence imaging to monitor formation longitudinally in the same cultures, supporting its use as a high‐throughput platform for drug discovery.^[^ ^157^ ^]^ However, although these stains indicated drusen‐associated proteins, mineral‐specific characterization was not performed,^[^ ^157^ ^]^ which should be a core consideration of any proposed AMD drug‐discovery platform and, more broadly, for any platform intended to mimic diseases where calcification occurs.

The success of cell models replicating drusen^[^ ^96^ ^]^ (particularly its mineral composition), without requiring osteogenic additives, highlights that calcification can occur in specific pathological contexts independently of osteogenic cues provided through the culture medium. Perhaps the successes of several drugs in slowing AMD progression^[^ ^158^ ^]^ are related to this mechanistic difference.

### 3.4 Pathological Calcification and the Impact of Cell Culture

As mentioned earlier, pathological calcification is a hallmark of numerous and often unrelated diseases. Still many researchers implicate transdifferentiation of calcifying cells into osteoblast‐like phenotypes as a central mechanism. This has prompted the widespread use of BGP‐based osteogenic induction media, typically containing ascorbic acid and dexamethasone too, in cell models to mimic calcification, particularly in contexts such as renal, vascular, joint, and breast calcifications.

Osteoblast‐like phenotypes are found in many calcified tissues, but the “osteogenic markers” currently analyzed including OPN, TNAP, and even Runx2 are insufficient to describe a fully developed osteoblast phenotype. These markers are also expressed in a variety of noncalcified tissues, limiting their specificity with the exception of retinal calcification in macular degeneration, where such markers are notably absent.^[^ ^156^ ^]^ The successful replication of drusen formation without osteogenic additives^[^ ^96^ ^]^ underscores the need to account for disease and tissue‐specific differences in calcification mechanisms.

This mechanistic divergence offers opportunities to refine therapeutic approaches. Work on genetic skin disorders suggested a causative role of TNAP in skin calcification.^[^ ^154^ ^]^ Indeed, the overexpression of TNAP under a skin‐specific promoter caused universal calcification of the skin's collagen in a mouse model.^[^ ^159^ ^]^ Whether or not inhibitors of alkaline phosphatase can prevent calcification in the context of increased TNAP activity is the topic of preclinical studies.

### 3.5 Implants—Materials That Prevent Pathological Calcification

The same concept of tailoring surface physical and chemical properties to enhance material adhesion to orthopedic implants and promote calcification has also been applied, in reverse, to inhibit calcification on implant surfaces where its prevention is critical to success.^[^ ^12^ ^]^

#### 3.5.1 Heart Valve Replacements

Among all implanted medical devices, replacement heart valves are the most recognized example where pathological calcification compromises function. An estimated 275 000 heart valve replacement procedures are performed each year, most involving valves made from animal pericardium (bioprosthetic valves).^[^ ^160^ ^]^ However, approximately half of all bioprosthetic valves fail within 15–20 years of surgery, with calcification being one of the leading causes of failure.^[^ ^12^ ^]^

These tissues undergo extensive chemical processing to eliminate cells and remove soluble macromolecules, including proteins, RNA, and DNA, that may otherwise act as proinflammatory‐damage‐associated molecular patterns. Following the extraction of soluble compounds, the remaining ECM macromolecules, mostly collagen, are cross‐linked using aldehydes, with glutaraldehyde being the preferred chemical.^[^ ^12^ ^]^

Pericardial tissue and xenogeneic pig heart valves are the most commonly used materials in commercial bioprosthetic valves.^[^ ^12^ ^]^ Most investigations of calcification in these valves have focused on evaluating how chemical modification affects the material's propensity to induce calcification. For instance, glutaraldehyde‐treated porcine aortic cells calcified in high‐calcium media but not in control media, depleting calcium ions in the process.^[^ ^161^ ^]^ Other mechanistic explanations have been proposed to clarify the factors contributing to calcification in glutaraldehyde‐fixed valves including alterations in the composition of the organic matrix, mechanical stress applied to the valve, and mechanisms related to cellular damage.^[^ ^12^ ^]^

Emerging strategies to mitigate valve calcification include the development of acellular‐tissue‐engineered vascular grafts, which have been successfully implanted in animal models such as lambs and baboons.^[^ ^162^ ^]^ Additionally, research has focused on biological inhibitors of mineral growth. In vascular calcification models, osteopontin was shown to bind to growing apatite crystals, inhibiting further growth and thus calcification.^[^ ^163^ ^]^ This finding spurred interest in using osteopontin to pretreat animal tissue valves as an anticalcification strategy.^[^ ^12^ ^]^

Further complicating the issue, osteoblast‐like cells have been identified in calcified bioprosthetic valves,^[^ ^164^ ^]^ and 3D‐printed valve models are now being used to investigate cell infiltration, mineralization, and osteogenic transdifferentiation^[^ ^165^ ^]^ including models that mimic physiological fluid flow and pressure.^[^ ^137^ ^]^ These works underscore the importance of understanding both material and cellular contributors to calcification in implanted devices.

#### 3.5.2 Intraocular Lenses

Cataract surgery to correct the clouding of the eye's lenses is the most commonly performed surgical procedure worldwide, with over 20 million operations conducted annually.^[^ ^166^ ^]^ This procedure replaces an opacified lens with a prosthetic lens, typically made from hydrogel or silicone.^[^ ^167^ ^]^ The most frequent complication, affecting ≈30% of patients within 7 years postsurgery, is the opacification of the prosthetic lens.^[^ ^166^ ^]^ A primary cause of this opacification is calcification on the surface of the implanted lenses.^[^ ^166^ ^]^ This condition often necessitates lens replacement, which increases the risk of surgical complications.^[^ ^166^ ^]^

Calcification is believed to result from disruptions in ion concentration or the introduction of impurities into the aqueous humor during cataract surgery.^[^ ^166^, ^167^ ^]^ Notably, an osteogenic process has also been proposed as a contributing factor.^[^ ^168^ ^]^ Osteoblast‐related proteins were found on calcified intraocular lens surfaces, and eye lens epithelial cells have been observed to transdifferentiate in vitro into an osteoblast‐like phenotype when exposed to osteogenic additives.^[^ ^168^ ^]^ More recent calcification models of lens calcification have investigated promoters of the transdifferentiation in specific pathologies such as diabetic cataracts. In an in vitro model, lens epithelial cells cultured with osteogenic additives in a high‐glucose medium appeared to accelerate calcification via hypoxia‐inducible factor signaling, which upon silencing attenuated the response.^[^ ^169^ ^]^

#### 3.5.3 Ureteral Stents

Ureteral stents are devices used to maintain the channel between the kidneys and bladder, which can become obstructed by tumor growth or kidney stones.^[^ ^170^ ^]^ Typically made of silicone or other soft plastics, over 1.5 million stents are implanted annually.^[^ ^170^ ^]^ However, more than 80% fail within months,^[^ ^170^ ^]^ often due to blockages caused by encrusted bacterial biofilm formation on the urine‐facing surface.^[^ ^170^ ^]^ These biofilms frequently contain calcium oxalate and phosphate minerals.^[^ ^171^ ^]^

Given the high volume of stent implantations and their significant failure rate, numerous new designs have been developed.^[^ ^170^ ^]^ These include using more flexible and strong materials such as advanced silicones and copolymer blends.^[^ ^170^ ^]^ In tandem, antimicrobial coatings, incorporating silver or drug‐eluting hydrogels, have been explored to inhibit bacterial growth via calcification models. These have, for example, shown how natural polymers such as propolis can reduce bacterial adhesion and proliferation on stent surfaces which corresponded to reduced calcification in vivo.^[^ ^170^, ^172^ ^]^ Hydrogel coatings are also being investigated to reduce friction during implantation, therefore minimizing inflammation, a contributing factor to stent calcification.^[^ ^170^ ^]^

Microbial cell calcification models have shown that stagnant flow through stents increases biofilm mineral content, prompting the development of designs that improve flow dynamics, most recently being investigated using advanced “stent‐on‐a‐chip” organoid models.^[^ ^173^ ^]^ In these microbial calcification models, BGP readily promotes bacteria observed within stent biofilms to produce apatite.^[^ ^173^, ^174^ ^]^

## 4 Cell Calcification Models—The Main Players

### 4.1 The Substrate

The substrate is one of the most powerful determinants of calcification in cell culture models, influencing both mineral deposition and osteoblast‐associated protein expression. Most cell calcification models are performed on plastic as 2D cultures, some employing scaffold materials that better represent implants and tissue. These studies show that materials cause different protein expressions and calcification in cells.^[^ ^59^, ^75^ ^]^ Apatite substrates, and to a lesser extent metal substrates, often cause calcification and bone protein expression to a higher degree than plastic.^[^ ^59^, ^75^ ^]^ Indeed, some apatite minerals release phosphate into the medium, such as calcium‐deficient hydroxyapatite,^[^ ^76^, ^77^ ^]^ while others deplete it, such as hydroxyapatite, α‐ and β‐tricalcium phosphate,^[^ ^77^ ^]^ with the general trend being that culturing on apatite enhances calcification.

Other than the chemical surface composition, topography has a strong effect on the inducement of cell calcification. Optimum roughness ranges for calcification and osteoblast‐related protein expression have been proposed^[^ ^78^ ^]^ and above this threshold, calcification appears to decrease.^[^ ^78^, ^79^ ^]^ Some works have, however, suggested that microroughness either does not change the expression of osteoblast‐related proteins^[^ ^80^ ^]^ or that it has an inhibitory effect.^[^ ^82^ ^]^ Others suggest that osteoblast‐related protein expression increases in response to microroughness, but calcification does not.^[^ ^81^ ^]^ These discrepancies may result from the method of microroughness measurement, which typically ignores feature overhangs, underestimating true surface area^[^ ^175^ ^]^ and disguising the importance of specific features.^[^ ^84^, ^86^ ^]^ For example, semirandomly arrayed pits 100 nm deep on plastic caused stem cells to express osteoblast‐related proteins in the absence of osteogenic media additives^[^ ^84^ ^]^ and single‐cell cultures showed that certain substrate shapes influence stem cell expression of osteoblast‐related proteins.^[^ ^86^ ^]^

Another factor affecting cell calcification is the stiffness of the material where cells are cultured. If gels are tailored to mimic the stiffness of osteoid, then osteoblast protein expression in cultured stem cells dramatically increases.^[^ ^58^ ^]^ Two other important factors are access to ions and flow. The latter refers to the rate by which fluid can flow through porous substrates and depends on the available surface area (which is typically far higher in 3D organoid cultures than in 2D), but is also sensitive to pore size and relative density of the porous substrate.^[^ ^176^ ^]^ Cell calcification models have, however, indicated that whenever ion depletion occurs, osteoblast protein expression increases.^[^ ^77^ ^]^ Ion depletion may be more likely to occur in regions of decreased fluid mobility or stagnant zones and has been suggested to be a cofactor for implant calcification in vivo.^[^ ^11^ ^]^

Another parameter rarely remembered is the substrate interaction with the culture media. If the substrate surface has an affinity for osteogenic media additives, then calcification can dramatically increase.^[^ ^59^, ^60^ ^]^ Moreover, acellular culture of calcium‐deficient apatite substrates shows that ion exchange between the substrate and media varies, depending on the culture media and osteogenic additives used.^[^ ^76^ ^]^ Further, culturing osteoblasts and stem cells on apatite substrates and on glass coverslips adjacent to an apatite substrate showed that osteoblast marker proteins dramatically increased in both cases.^[^ ^77^ ^]^

Scaffold choice is critical in cell calcification models, influencing outcomes through material properties, surface chemistry, topography, stiffness, and ion concentration. Apatite and metal substrates often enhance calcification and protein expression compared to plastic, while microroughness and stiffness also play significant roles. Ion availability and substrate–media interactions further modulate calcification, highlighting the need for careful consideration of these factors to create accurate and effective calcification models.

### 4.2 The Media

The cell culture medium composition primarily determines whether calcification will occur. Indeed, ordinary base media is, accepting the subtleties of its calculation,^[^ ^177^ ^]^ supersaturated with respect to apatite^[^ ^47^ ^]^ and additives used in nearly all cell calcification models increase this further through the addition of BGP, or inorganic calcium and phosphate sources. This simple fact, often either unknown, forgotten or overlooked, should be strongly highlighted because it may cause false positive results related to calcification. Researchers might be led to mistakenly report calcification due to cellular behavior, when in fact the calcification observed is simply a result of precipitation from the media. As summarized in Figure 2, ion supersaturation and protein–mineral complexation underpin mineral formation pathways in vitro and hence any observation must be interpreted in the context of solution chemistry.

Moreover, the base medium itself also presents challenges for reproducibility. For example, Dulbecco's Modified Eagle Medium (DMEM), one of the most commonly used base mediums, is designed to maintain a pH of 7.2–7.4 in an atmosphere containing 10% CO_2_ (ref. [178]). However, many cell models using DMEM incubate in 5% CO_2_, causing a pH of 7.6 or higher in the culture^[^ ^179^ ^]^ (unless the concentration of bicarbonate is altered to compensate for the variation in CO_2_, which, on the other hand, can itself alter cell behavior). Such a small deviation cannot be detected by the normal phenol red indicator in the media, but some reports suggest that it may impact mineral formation and reduce TNAP activity by ≈60% (ref. [45]).

Another critical consideration in media formulation is the use of serum, which is routinely added to cell cultures to provide essential growth and attachment factors, amino acids, and other components necessary for cell viability. One of the most abundant mineral‐binding proteins in serum is fetuin‐A—particularly in fetal bovine serum (FBS), from which its name is derived. Fetuin‐A, along with other proteins capable of forming protein–mineral complexes, can inhibit apatite precipitation such that mineral formation is rarely observed in control cultures supplemented with serum.^[^ ^47^ ^]^

Another factor that may impact mineral formation is that the considerable batch‐to‐batch variability in sera concentrations of substances such as fetuin‐A, TNAP, pyrophosphate, magnesium, and phosphate which all greatly affect mineral formation from solution.^[^ ^42^, ^47^, ^180^ ^]^ This variability has led to a practice of searching for serum batches that “work.”^[^ ^181^ ^]^ That is, promoting calcification when the goal is to induce calcification in the cell model while conversely selecting batches that have the opposite effect when calcification is not desired^[^ ^181^ ^]^ without considering what makes a serum batch “work” or not.

Adding to the variability, the concentration of serum used in calcification models can differ between studies—most commonly at 10% v/v, but in some cases reaching as high as 20%. Some vascular calcification models with no specific calcification additives have reported osteoblast‐like phenotypes and calcification^[^ ^182^, ^183^ ^]^ that could have occurred because of the large amount of serum added, up to 20%.

If the use of base media and serum are not already controversial enough regarding their effects on calcification in cell models, additives introduce an entirely new complexity. Unsurprisingly, there is no clear consensus in the literature about the impact of media additives. Some studies suggest, for instance, that BGP induces both apoptosis and calcification,^[^ ^184^ ^]^ while others report that it induces calcification without triggering apoptosis.^[^ ^185^, ^186^ ^]^ The simple solution to this conundrum may be that some cells, such as SaOS‐2, are rich in TNAP and convert BGP to phosphate in a short time, causing acute phosphate toxicity. In these cells, reducing BGP usually helps survival and prevents catastrophic precipitation of calcium phosphate onto dead and dying cells. Additionally, it has been suggested that inorganic calcium and phosphate salts do not cause apoptosis but do promote calcification.^[^ ^187^ ^]^ Indeed, some research indicates that calcification itself induces apoptosis, which in turn promotes further calcification, regardless of the presence of BGP or calcium and phosphate salts.^[^ ^183^ ^]^ Finally, in vascular smooth‐muscle cells that have transdifferentiated to an osteoblast‐like phenotype, calcium and phosphate salts induce extracellular vesicles with reduced TNAP, mimicking hyperphosphatemia (a common symptom of chronic kidney disease), whereas BGP induces TNAP‐rich vesicles,^[^ ^188^ ^]^ yet BGP is still used in some hyperphosphatemia models.^[^ ^189^ ^]^

At present, the effects of media, serum, and additives are not fully considered by many researchers, and more controlled and standardized studies must be conducted if we aim to determine which factors truly induce calcification in cell culture models. The use of calcification models is so widespread across different fields that a combined effort to standardize and better understand the conditions for calcification is challenging to achieve. Moreover, and perhaps unfortunately, calcifying models have existed for so long that it is now well‐known how to induce calcification in practically any cell culture. This knowledge in some ways undermines the true objective of understanding the mechanisms of calcification, as many studies simply aim to prove whether their new material can calcify or not, a result that can be easily attained by adapting the culture conditions. Such studies fail to provide meaningful information about how the material or conditions being investigated truly affect the cells.

### 4.3 The Cells

Adding to the discrepancies associated with media variability, another substantial variable in cell calcification models is the cells themselves.

It is well established from pathological cell calcification models that many types of cells, including those from the heart, brain, and eye, can transdifferentiate into an osteoblast‐like phenotype and undergo calcification. Within the natural osteoblast lineage, the process begins with stem cells. These progenitor stem cells follow a specific developmental pathway, differentiating into osteoblasts under appropriate conditions.^[^ ^190^ ^]^ In culture, stem cells from this lineage can be induced to differentiate into osteoblasts using the same media supplements that promote calcification in other cell types.^[^ ^190^ ^]^ The ability to differentiate into osteoblasts, chondroblasts, and adipocytes is in fact among the “minimal criteria” defining multipotent mesenchymal stem cells,^[^ ^190^ ^]^ also known as medicinal signaling cells and mesenchymal stromal cells.^[^ ^191^ ^]^ For multipotent stem cells to undergo osteogenic differentiation, a confluent monolayer is treated with a combination of dexamethasone, ascorbic acid, and BGP. Dexamethasone increases Runx2 activity through upregulating transcriptional coactivator with PDZ‐binding motif and mitogen‐activated protein kinase phosphatase 1 and through transcriptional activation mediated by four and a half LIM domains protein 2/β‐catenin.^[^ ^48^ ^]^ Increased collagen type I (Col1)/α2β1 integrin‐mediated intracellular signaling results from ascorbic‐acid‐induced increased Col1 secretion.^[^ ^48^ ^]^ The phosphate from BGP serves as a source for the phosphate in hydroxyapatite and in addition influences intracellular signaling molecules.

The ability of mesenchymal or “adult” stem cells to calcify poses an important dilemma for cell calcification models, since several studies have shown that these stem cells are present in “every vascularized tissue”^[^ ^191^, ^192^ ^]^ and are suspected to be present in practically every tissue.^[^ ^31^ ^]^ The presence of these calcifying cells suggests that a considerable amount of calcification observed in primary cell cultures may not only result from cell transdifferentiation, but also from the differentiation of stem cells within the culture.^[^ ^31^ ^]^ Moreover, the variation in calcification observed between cultures (even when using the same parameters, but different donors) could be caused by a difference in the concentration of mesenchymal stem cells between donors and tissues.^[^ ^192^ ^]^ The effects of mesenchymal stem cells (and similar cells^[^ ^31^ ^]^) in calcification models could be better compared by quantifying their abundance in the culture (**Table** **2**) and, where feasible, by isolating these cells and systematically varying their seeding ratios.

**Table 2:** Best practices and suggested reporting in cell calcification models to be used in conjunction with existing general cell culture guidelines.^[^ ^178^, ^195^ ^]^

Report and incubate at the specific % CO_2_ and humidity that the base medium was designed for, e.g., DMEM: 10%, M199: 5% (ref. [178]). Report base medium product catalogue number, manufacturer, and batch number. Cells can calcify in cultures with synthetic serum,^[^ ^185^ ^]^ so this must be used whenever possible as its exact constitution is known and should be reported. If using animal serum, test and report the alkaline phosphatase, calcium, and phosphorus contents. Also report product catalog number, manufacturer, and batch number. In all cases, use 10% v/v serum in the chosen base medium unless a calcification model niche explicitly requires otherwise. Use additives with defined stoichiometries, for example: β‐glycerophosphate pentahydrate. When modeling hyperphosphatemia, use calcium and phosphate salts as osteogenic additives and not BGP. If culturing with a gel, report its stiffness, average pore size, and relative density. When using primary cells, report the number of passages before culturing with calcification media. Also, quantify and report mesenchymal cell (and phenotypically similar cell^[^ ^31^ ^]^) abundance in these populations using defined protocols, such as staining or flow cytometry, for currently accepted marker proteins.^[^ ^31^ ^]^ Adhere to a stringent schedule for medium exchange and utilize freshly prepared calcification formulations, as prolonged storage in the refrigerator should be avoided. Use at least one mineral‐specific analysis technique, such as electron diffraction, to confirm the presence of mineral.

Still in connection with cells, another important factor to be considered is the age of the cell line. Within studies of the same cell line, conflicting results have been observed. For example, Hs27 cells, a skin fibroblast line, did not calcify in one study^[^ ^101^ ^]^ but did in another^[^ ^102^ ^]^ when cultured with the same media additives. Similar discrepancies have been reported in other cell lines, such as HeLa,^[^ ^99^, ^105^ ^]^ HEK293 (refs. [99, 105]), and MRC‐5 (refs. [98, 193]). Such discrepancies could be due to phenotypic and genetic drift after many passages. Indeed, a HeLa cell culture in one lab will likely have different properties to another lab.^[^ ^194^ ^]^ This drift is particularly important in calcification studies, as vascular cells have been shown to calcify to different extents depending on the number of passages.^[^ ^130^ ^]^ Typically, primary cells are used in calcification models between passages 2 and 5, but even between passages 1 and 4, TNAP activity across the cell population decreases significantly, reducing BGP‐induced calcification.^[^ ^130^ ^]^

Cell passage variation is a well‐known phenomenon and is undoubtedly one of the main parameters considered by any cell culture researcher.^[^ ^178^, ^195^ ^]^ However, it should be emphasized that small variations in passages, which may not typically have a significant effect on cells in cell culture studies unrelated to calcification, can still have a considerable impact on cell calcification models.

Perhaps even more important than cell passage number is the inherent heterogeneity of the cells used in calcification models. While heterogeneity is well recognized when using primary cells, it is often underestimated when using stem cells. Many researchers overlook the fact that stem cells are highly responsive not only to osteogenic additives but also the presence of different materials. These materials may, for instance, mimic the composition of bone, such as calcium phosphate ceramics,^[^ ^66^, ^77^ ^]^ or replicate the mechanical stiffness of bone, as observed in ceramics and metals,^[^ ^58^, ^176^ ^]^ and thus considerably induce the differentiation of stem cells to bone cells, even without any calcifying media or additive.

### 4.4 Characterization Methods

Albeit somewhat unexpectedly, a major source of variation in cell calcification models arises from the choice of calcification characterization method. The most common and inexpensive methods typically rely on optical staining techniques. While these stains are widely considered indicative of positive calcification results,^[^ ^190^ ^]^ they often yield false positives.^[^ ^196^ ^]^ Stains such as Alizarin^[^ ^196^ ^]^ and von Kossa^[^ ^197^, ^198^ ^]^ can indicate the presence of calcification when no mineralized material is present, by simply highlighting the accumulation of calcium and phosphate ions.

Therefore, detecting calcification forming in a cell culture is contingent on how the calcification is characterized, and this can be done via a combination of techniques. Binderman et al.’s study,^[^ ^39^ ^]^ the first to demonstrate bone cell culture calcification, set a precedent for multimodal characterization. This work applied von Kossa and alkaline phosphatase stains, observed using bright‐field and phase contrast light microscopes. In addition to these traditional staining methods, they also used electron microscopy and monitored calcium radioisotope uptake.^[^ ^39^ ^]^

In recent years, it has become increasingly common to report calcification in cell culture using simple stains combined with protein expression assays. A positive result from these stains and the presence of specific proteins is typically interpreted as evidence of calcification and transdifferentiation into osteoblast‐like cells.^[^ ^190^ ^]^ For example, TGF transforming growth factor beta 1 is often observed to cause the upregulation of osteoblast‐related proteins in cells^[^ ^199^ ^]^ and its observation in association with Alizarin Red staining has suggested the presence of calcified nodules in a culture.^[^ ^199^ ^]^ However, comparative Raman microscopy on similar cultures showed no mineral in these nodules,^[^ ^196^ ^]^ highlighting the importance of using mineral‐specific analysis techniques.

Even though the use of staining alone for confirmation of calcification, which is premised on the staining of its mineral component, poses a clear problem for cell calcification models, another overlooked problem in the field is protein expression reports. The expressions of proteins associated with osteoblast‐like calcification, such as Runx2 and TNAP, are frequently analyzed in calcification models. Upregulation of these proteins is often observed in cultures exposed to BGP, ascorbic acid, and dexamethasone. However, the cellular activity of some proteins, such as TNAP, can be substantially affected by the activity of proteins in the serum, resulting in cultures that exhibit high osteoblast‐like protein activity but minimal mineralization.^[^ ^180^ ^]^ The same can be said of other markers, such as osteocalcin and bone sialoprotein.

Finally, it is important to remember that not all calcification is the same. It is intuitive to state that bones and teeth are not formed by the same cells, nor do they contain the same material, with bone being composed of poorly crystalline apatite and enamel being composed of highly crystalline apatite.^[^ ^1^ ^]^ Unsurprisingly, but definitely overlooked is the fact that this distinction also applies to different forms of calcification, particularly in pathological calcifications, such as those formed in the vascular system,^[^ ^3^ ^]^ the eye,^[^ ^156^, ^200^ ^]^ and the brain.^[^ ^6^ ^]^

These variations underscore the need for careful and context‐specific characterization in cell‐based calcification models. A complete assessment cannot rely solely on staining or gene expression. Critically, no single method can determine whether the mineral produced in vitro replicates the calcification found in the corresponding tissue in vivo. Therefore, a multimodal approach is essential.

Comprehensive characterization should combine traditional staining and molecular assays with high‐resolution physicochemical techniques. Methods such as scanning or transmission electron microscopy, Raman or infrared spectroscopy, and X‐ray diffraction can provide insights into mineral morphology, atomic structure, and chemical composition. Combining modalities visually often aids the interpretation of calcification features.^[^ ^201^ ^]^ Only through this level of detailed analysis can researchers accurately assess whether a cell model is faithfully replicating a specific calcification process, be it physiological or pathological.

## 5 Conclusion

**Figure 3:**
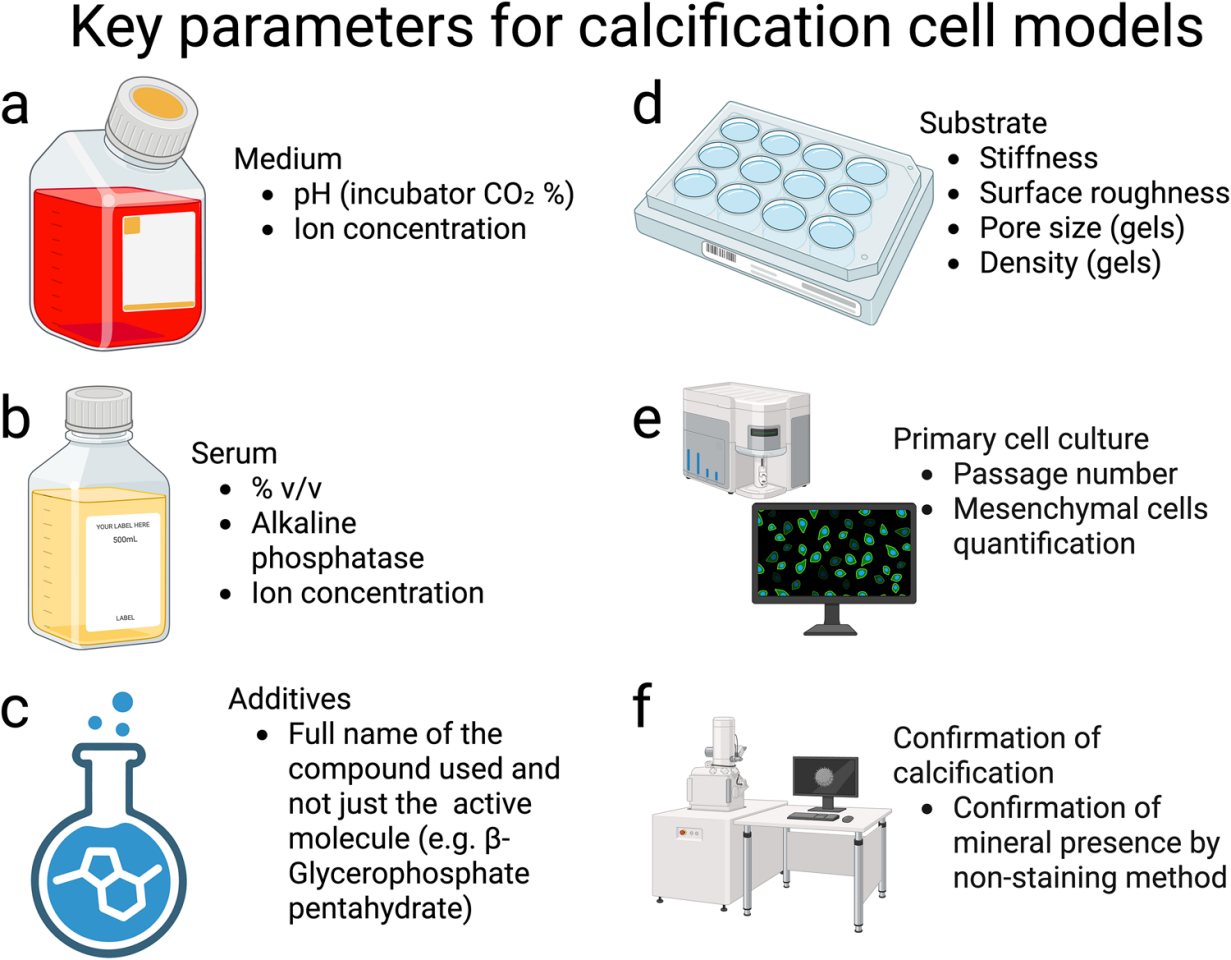
Main parameters for best practices of cell calcification models that must be reported and controlled. a) Medium composition, with particular attention to pH, CO_2_ concentration, and ion concentration in the basal medium. b) Serum composition and concentration in the medium, with particular attention to alkaline phosphatase and ion concentration. If possible, use synthetic serum to avoid inconsistencies between experiments. c) Report the full list and concentration of additives added to the media. d) Report properties of the substrate, such as its stiffness, pore size distribution, and density. e) For primary cell cultures, quantify and report the amount of mesenchymal stem cells present in the culture. f) Confirm the presence of mineral in the culture by at least one non‐staining method such as electron microscopy, X‐ray diffraction, or spectroscopic methods (Raman or infrared spectroscopy). Figure created with BioRender.com.

Calcifying cell models cover a wide range of studies with diverse, and often contradictory, objectives, such as promoting calcification or preventing it. Regardless of the specific aim, their ultimate goal is to understand or replicate phenomena that occur in vivo. In this sense, cell calcification models should be scrutinized regarding physiological relevance and reproducibility (see Table 2 and **Figure** **3** for suggestions on best practices for cell calcification models). These models exist within one of the most truly interdisciplinary fields, where a deep understanding of chemistry, biology, and medicine is fundamental. A cross‐disciplinary effort is required for making significant advancements in these models, as well as acknowledging the many unknowns posed by factors and methods, and the several tools available to address them.

Significant differences exist in biological calcification processes. Such differences should be acknowledged and celebrated, as they hold the key to developing targeted therapies that do not adversely affect other calcification processes in an organism. For example, a treatment designed to prevent vascular calcification ideally must not inhibit normal bone growth and remodeling, and conversely, a bone growth promoter should not induce vascular calcification. Therefore, it is fundamental that calcification cell culture users pay close attention to the sometimes subtle differences between the different models, as they could suggest different calcification mechanisms.

Though not always recognized as such, calcifying cell models have far‐reaching applications. They are essential for advancing our understanding of mineralized tissue biology and for studying diseases that affect nearly every human organ—given that pathological calcification can occur across virtually all tissues. As one of the most broadly used classes of cell culture models in biomedical research, ensuring that these systems are designed, performed, and reported with reproducibility and rigor is more important than ever. Only then can they provide meaningful, translatable insights into the complex process of calcification.

## Conflict of Interest

The authors declare no conflict of interest.
